# Evolution and intelligent design in drug development

**DOI:** 10.3389/fmolb.2015.00027

**Published:** 2015-05-21

**Authors:** Roman V. Agafonov, Christopher Wilson, Dorothee Kern

**Affiliations:** Howard Hughes Medical Institute and Department of Biochemistry, Brandeis UniversityWaltham, MA, USA

**Keywords:** drug design, evolution, cancer drugs, protein kinases, conformational selection and induced fit, Gleevec

## Abstract

Sophisticated protein kinase networks, empowering complexity in higher organisms, are also drivers of devastating diseases such as cancer. Accordingly, these enzymes have become major drug targets of the twenty-first century. However, the holy grail of designing specific kinase inhibitors aimed at specific cancers has not been found. Can new approaches in cancer drug design help win the battle with this multi-faced and quickly evolving enemy? In this perspective we discuss new strategies and ideas that were born out of a recent breakthrough in understanding the molecular basis underlying the clinical success of the cancer drug Gleevec. An “old” method, stopped-flow kinetics, combined with old enzymes, the ancestors dating back up to about billion years, provides an unexpected outlook for future intelligent design of drugs.

## The beauty and curse of protein kinases

Why are we more sophisticated than a yeast cell? One of the reasons is protein kinases, that exploded both in numbers (more than 500 in humans compared to 130 in yeast) and sophistication with the development of multicellularity (Richter and King, 2013). The evolution of specialized kinases enabled complex regulatory networks in higher organisms thereby providing a huge evolutionary edge. However, a crack in this machinery as little as a single point mutation in a kinase can cause cancer—an Achilles heel that has elevated protein kinases into the number one drug target of the twenty-first century (Cohen, 2002; Cohen and Alessi, 2013; Wang et al., 2014). The stringent requirements for catalyzing a chemical reaction that uncatalyzed would take about 7000 years (Stockbridge and Wolfenden, 2009; Kerns et al., 2015) resulted in a strong conservation of the active sites, which have thus been extensively targeted in cancer drug development. Unfortunately, inhibitors targeting the ATP binding site tend to be unselective due to this active site conservation, leading to unwanted side effects. The popularity of the field of protein kinase inhibition as well as alternative strategies such as inhibition of substrate binding and protein interaction sites is best reflected by a number of recent reviews (Wang et al., 2014 and a special issue in ACS Chemical Biology, 2015). In addition, new high-throughput assays are being constantly developed to facilitate screening of the compounds (Acker and Auld, 2014), however, the major goal of the pharmaceutical industry to develop specific kinase inhibitors remains a daunting challenge.

## The wonder drug of the century

Gleevec is an exception, as it has great specificity for the onco-protein BCR-Abl (Capdeville et al., 2002; Cohen et al., 2002). The BCR-Abl fusion protein results from reciprocal translocation between chromosome 9 and chromosome 22, widely known as the Philadelphia translocation, leading to a constitutively active kinase (Rowley, 1973; Daley and Baltimore, 1988). Gleevec was approved by the FDA for clinical use in 2001, and has proven to be remarkably successful in treating chronic myeloid leukemia (CML) and gastrointestinal stromal tumors. Its success generated tremendous enthusiasm in the scientific community and even general public, after the reports about “new ammunition in the war against cancer” and its outstanding effectiveness were picked up by the media (Lemonick and Park, 2001; Newsweek, 2001; Wade, 2001). Gleevec was viewed as a “proof of principle drug,” which showed the possibility of rational design of an inhibitor that would specifically target a kinase of interest. Unfortunately, tireless efforts aimed at understanding the molecular mechanisms of Gleevec's selectivity over the last 20+ years were mostly unsuccessful, and the original expectations of a steady stream of new therapeutics emerging from basic research turned out to be overoptimistic. As reviewed recently (Cohen and Alessi, 2013; Wang et al., 2014) since Gleevec's triumph, approximately 20 new kinase inhibitors were developed and entered clinical use. This is a rather small number considering that there are more than 500 human kinases and multiple inhibitors are needed for each of them to combat the inevitable mutations that lead to drug resistance. A fundamental pitfall in drug development is a lack of understanding of the detailed biophysical mechanisms that make inhibitors successful.

## Conformational selection and the famous “DFG-loop”

**Figure 1:**
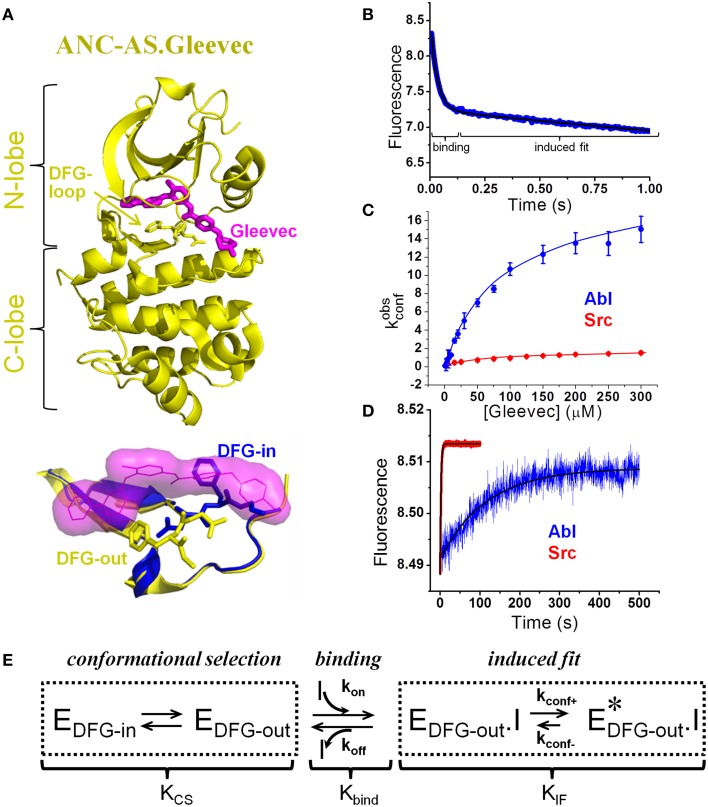
**Novel model of Gleevec binding to tyrosine kinases with quantification of individual steps**. **(A)** Top: Crystal structure (4CSV) (Wilson et al., 2015) of last common ancestor of Src and Abl (ANC-AS) bound to Gleevec (magenta); the DFG loop is shown in stick. Bottom: DFG-loop in the -in (2SRC) and -out (4CSV) conformation is shown with Gleevec bound (magenta surface). Only the DFG-out conformation is compatible with Gleevec binding. **(B–D)** Binding and dissociation kinetics of Gleevec to Abl and Src measured by stopped-flow fluorescence (for details see Agafonov et al., 2014). **(B)** Gleevec binding to Abl at 5°C is biphasic with the fast phase corresponding to the physical binding step and slow phase corresponding to the induced fit step. Blue – experimental data, black – double-exponential fit. **(C)** Dependence of k^obs^_conf_ [observed rate of the induced fit step, see scheme in **(E)**] on Gleevec concentration. **(D)** Dissociation kinetics of Gleevec from Abl and Src measured by dilution of enzyme-Gleevec complexes, which determines the k_conf-_ rate constant [see scheme in **(E)**]. **(E)** Gleevec binding scheme showing three distinct steps: conformational selection step, physical binding of the drug to the binding competent state, and the following conformational transition (induced fit). Equilibrium constants corresponding to each step (K_cs_, K_bind_, and K_IF_) determine the overall binding affinity $({\text{K}}_{\text{D}}):{\text{K}}_{\text{D}}=\frac{({\text{K}}_{\text{CS}}+\text{1})\;•\;{\text{K}}_{\text{bind}}\;•\;{\text{K}}_{\text{IF}}}{\left(\text{1}+{\text{K}}_{\text{IF}}\right)}$.

In the search for the physical determinants of Gleevec selectivity, the DFG – loop (Asp-Phe-Gly), a 100% conserved element in the kinome (Figure 1A), stood out as a structural feature that differs between kinases that bind Gleevec tightly or weakly. In the x-ray structure of Abl, this loop adopts an “out” conformation in both the apo and Gleevec-bound protein, while in the closest homolog and weak binder Src kinase it occupies a binding-incompetent “in” conformation in the apo protein that would have to move into the “out” position to accommodate the drug (Xu et al., 1997; Schindler et al., 2000; Nagar et al., 2003; Seeliger et al., 2007). These structures, together with the fact that the active conformations look too similar to provide selectivity, shifted attention toward structural determinants of inactive conformations.

It was hypothesized that the preferential occupancy of the DFG-out state by Abl but not Src is the primary source of Gleevec selectivity. This model of an equilibrium between binding-incompetent (DFG-in) and competent state (DFG-out) (K_CS_) being the source for differential drug affinities is a classical conformational selection mechanism (Cowan-Jacob et al., 2005; Dar et al., 2008; Shan et al., 2009; Aleksandrov and Simonson, 2010; Lovera et al., 2012; Lin and Roux, 2013; Lin et al., 2014) that has recently gained popularity in biology (see the special issue of Biophysical Chemistry and references within) (Biophysical Chemistry, 2014) (Scheme in Figure 1E). This hypothesis was further substantiated by the observation that less selective inhibitors such as Dasatinib do not differentiate between “in” and “out” conformations of the DFG-loop.

The elegance of this hypothesis, the direct observation of two different states of the DFG-loop in crystal structures and the excellent fit to the “expected model” of drug selectivity resulted in a wealth of literature focusing on this aspect of protein dynamics. A variety of approaches, both experimental (Vogtherr et al., 2006; Vajpai et al., 2008) and computational, were taken to quantify the free energy profile of the DFG-loop dynamics (Levinson et al., 2006; Aleksandrov and Simonson, 2010; Lovera et al., 2012; Lin and Roux, 2013; Lin et al., 2014; Meng et al., 2015). However, experimental studies of DFG-loop equilibrium in solution were complicated by high dynamics of this loop hampering quantification of this equilibrium. Some computational reports seemed to quite impressively quantitatively recapitulate the experimentally observed Gleevec affinities for the different kinases (Lin and Roux, 2013; Lin et al., 2014) despite the widely acknowledged current computational limitations for accurate energy calculations (Shaw et al., 2010; Piana et al., 2011; Lindorff-Larsen et al., 2012). Other computational studies were contradictory, and results varied depending on the methodology used. Despite the lack of direct experimental observation of the DFG-loop equilibrium, the DFG-loop hypothesis underlying selectivity became so popular that all active site kinase inhibitors were classified as class I (binding to both DFG-in and -out conformations) and class II (binding exclusively to the DFG-out state).

Although large screens hinted at a trend that class-II inhibitors may be more selective, many counterexamples of selective type I and promiscuous type II inhibitors were observed (Davis et al., 2011; Treiber and Shah, 2013). These data suggested that the DFG-loop may not be as essential for selectivity as initially thought. Paradoxically, despite its logical appeal, the DFG-loop conformational selection model did not lead to new highly selective kinase drugs. What is missing?

## Old fashioned?

A surprising breakthrough came from an unexpected direction. A new method of molecular time-travel back to the origin of these kinases and resurrection of their evolutionary trajectories into the modern kinases delivered the mechanism of Gleevec selectivity. Ironically, not only the resurrected enzymes that provided the understanding were old, so was the technique that yielded the answer. Stopped-flow kinetics, first described in the 1940s (Chance, 1940; Gibson et al., 1955) and often perceived as old-fashioned, has enormous potential when it comes to characterizing enzyme–drug interactions.

However the first hint for a new and unanticipated model came from following Gleevec binding to human Abl and Src by NMR, which revealed a slow conformational transition after drug binding that was different for the two kinases. Moreover, binding was sensed by residues far from the binding pocket indicating propagated conformational changes (Agafonov et al., 2014). Stopped-flow fluorescence experiments with modern Abl and Src (Agafonov et al., 2014) delivered quantification of the steps observed in the NMR experiments.

Contrary to the previously explored models, the dominant role in Gleevec's selectivity belongs to the conformational transitions in the kinase-drug complex (induced fit, Figure 1E), and not to the DFG-loop conformational selection or the physical binding step. These induced fit transitions are the slowest steps with the forward rate (k_conf+_) roughly 10 times faster in Abl compared to Src (Figure 1C). The rate of the reverse step, k_conf−_, measured by dilution experiments, is 70-fold slower in Abl (Figure 1D), leading to a 700-fold difference in the overall equilibrium (K_IF_) (Figure 1E). Because of simple principles of coupled equilibria, this 700-fold shift of the induced fit step equilibrium results in a 700-fold increase in the overall affinity for Gleevec, therefore accounting for most of the observed 3000-fold difference [the remaining four-fold difference comes from the DFG-loop conformational selection (see below)]. The actual binding step to the two kinases is nearly identical highlighting the limited usefulness of docking studies that play a prominent role in the current computational efforts in drug design. This “numbers-game” from the stopped-flow experiments delivered a new mechanism that quantitatively accounts for the long-known difference in kinase affinities for Gleevec and hence answers the long-standing question of specificity (Agafonov et al., 2014).

Inspired by the new findings for Gleevec we advocate that the full energy profiles need to be considered, since the differences between kinases are rooted in the differences of the free energies of all states along the binding trajectory. The role of induced fit in substrate binding to enzymes for better substrate positioning for catalysis has been appreciated, however its experimental quantification is still not a commonly applied practice. Possible roles of induced fit for drug binding was also nicely discussed (Copeland, 2011), but its role in inhibitor affinity and selectivity remains undervalued. Notably, only the local rearrangements around the drug-binding pocket instead of long-range conformational transitions are often considered in rational drug design. Such long-range dynamics is, in fact, in play for the Gleevec specificity, as exposed by the ancestor resurrection.

## The devil is in the [atomistic] details

While a physical chemist might be satisfied having figured out the kinetic scheme with hard numbers that rationalize the different drug affinities, the structural biologist will ask: which residues are responsible for the different energy landscapes? This might appear easy—just start mutating residues in the weak binding Src to mimic Abl. However, in spite of a large number of tested substitutions, such efforts were not successful indicating that the underling mechanism for Gleevec selectivity is more complex than anticipated (Seeliger et al., 2007). This approach although tempting has the following unavoidable drawbacks. Many differences accumulated during divergent evolution result from neutral drift (substitutions that are neutral for function and thus are not under selective pressure), and basically represent noise, from which one needs to fish out the sequence changes linked to the property of interest. To make the mater worse, some amino acid changes only come into play in the background of other mutations – a phenomenon called epistasis (Depristo et al., 2005; Harms and Thornton, 2013; Boucher et al., 2014). As a consequence, simple sequence swaps between two modern enzymes don't work because they miss the effect of the corresponding evolution of the amino acid background.

As illustrated in Wilson et al. (2015), ancestral sequence reconstruction (ASR) can be a powerful tool to overcome this challenge. ASR is a rapidly developing method that allows the inference of now nonexistent ancestral sequences using the growing amount of sequence information available. This approach was already formulated more than 50 years ago by Pauling and Zuckerkandl (1963). Modern enzymes (even the ones close in structure) still often differ from each other by 100+ residues. Such divergence in combination with neutral drift and epistasis makes it virtually impossible to rationally analyze the sequence differences. Ancestral reconstruction kills two birds with one stone. First, the sequence differences between two ancestors (or an ancestor and a modern protein) are smaller than those between the two modern enzymes, which makes a productive analysis of sequences more probable. Second, swaps between ancestor and its “grand-grand-children” can indeed shed light into atomistic mechanisms since epistasis is naturally accounted for.

**Figure 2:**
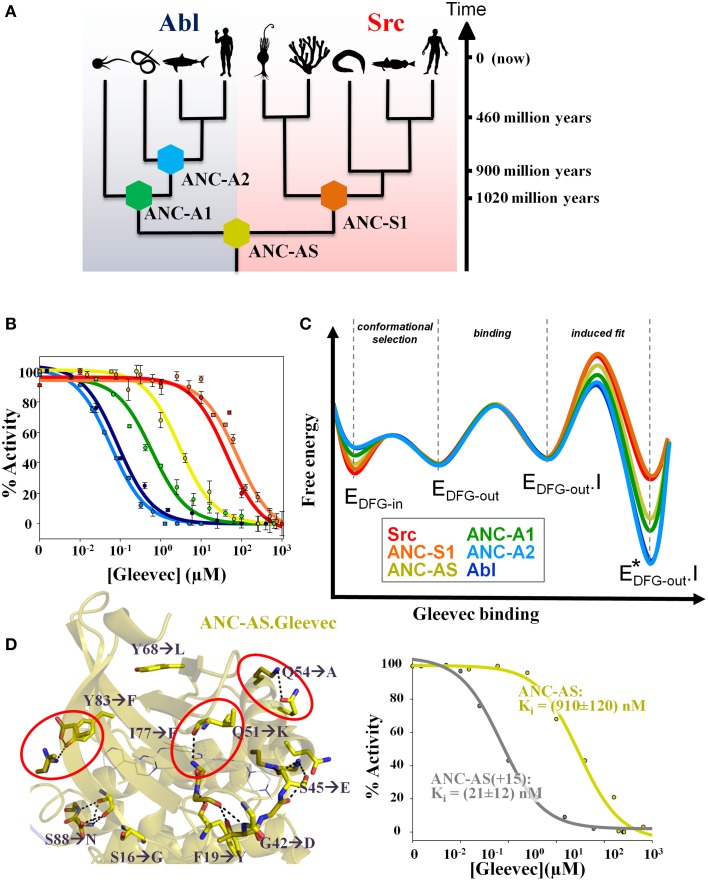
**Ancestral sequence reconstruction reveals the evolution of the energy landscape for Gleevec binding and identifies the residues responsible for Gleevec selectivity**. **(A)** Phylogenetic tree of Abl and Src families showing the reconstructed nodes. Timeline indicates approximate age of the reconstructed ancestors. The corresponding sequences including the alignment are given in Wilson et al. (2015) **(B)** Inhibition constants K_i_ for each kinase were determined from the activity versus drug concentration profiles showing a gradual change in Gleevec affinity from the weak binder Src to the tight binder Abl via the intermediate binder ANC-AS. Same colors are used as defined in **(A)**. **(C)** Schematic representation of the evolution of the Gleevec binding energy landscape based on data from Wilson et al. (2015). The major difference between kinases is in the induced fit step. Conformational selection step provides a minor contribution and physical binding step is nearly identical in all kinases. **(D)** Substitution of only 15 residues in the N-terminal lobe of ANC-AS (resulting in ANC-AS(+15)) guided by ancestral sequence reconstruction, structure, and biochemical analysis (Wilson et al., 2015) results in dramatic increase in Gleevec affinity (right panel). Ten of the amino acid changes from ANC-AS into the corresponding residues in Abl are indicated by arrows. A subset of these identified mutations disrupt hydrogen bonds (shown as dotted lines) that are present in weak binders (some highlighted by red circles) leading to an increase in kinase flexibility for the strong binders thereby enabling an efficient induced fit step. Some panels in Figures 1, 2 are adapted from Agafonov et al. (2014) and Wilson et al. (2015).

In the work of Wilson et al. (2015) a phylogenetic tree of 76 modern kinases from different families and organisms of non-receptor tyrosine kinases was reconstructed, and protein sequences corresponding to key evolutionary branching points were resurrected (Figure 2A). Remarkably, all reconstructed ancient enzymes, differing by up to 100 amino acids from anything you can find today in nature, are fully active! The common ancestor of Src and Abl (called ANC-AS) had an intermediate affinity for Gleevec that increased along the evolutionary branch leading to Abl and decreased along the Src branch (Figure 2B).

Combining ancestral reconstruction with their Gleevec binding kinetics and structure illustrates the evolution of divergent energy landscapes (Figure 2C). Of interest to drug designers, it indeed delivered the atomistic mechanism responsible for Gleevec selectivity. Fifteen amino acid differences (out of 146) were identified to encode Gleevec specificity for Abl (Figure 2D) (Wilson et al., 2015). Their role in the induced fit step can now be rationalized structurally including stabilizing effects on drug–protein interaction and tuning differential flexibility via H-bonds remote from the drug-binding site (Figure 2D) (Wilson et al., 2015). So indeed long-range dynamics and epistasis are in play for Gleevec binding as first seen in the NMR studies (Agafonov et al., 2014) and hinted by the unsuccessful early swop approach (Seeliger et al., 2007).

Interestingly, the same residues correlated well with several resistant mutations found in patients who developed Gleevec resistance (Wilson et al., 2015). In other words, current evolution appears in these “dynamic hotspots,” and the rationalization of the underlying atomistic mechanism for Gleevec resistance might help in designing drugs that overcome this detrimental evolution of cancer cells.

## New tool in biophysics—ancestral sequence reconstruction (ASR)

The reader should wonder why an evolutionary approach is useful to solve a mechanism of a modern-day, man-made molecule? Obviously Abl did not evolve to bind Gleevec and be “strangled” by it! Rather, Gleevec accidently took advantage of differences in kinase regulation created by divergent evolution. While kinases are similar in their turnover rates upon activation, they vary drastically in their regulatory mechanisms. Such evolution of regulation became necessary with the developing of multicellularity and increasingly complex signaling cascades. Although in the case of Gleevec phylogenetic considerations were not part of the design, and overlap between Gleevec's selectivity and evolution of regulation was coincidental, we propose that targeting the unique energy landscapes underlying the regulatory features of a kinase of interest can be a powerful strategy for developing new selective inhibitors.

Evolution is rooted in the most fundamental process of random mutations, and driven by selection for better fitness. In light of this, the weird link between Gleevec selectivity and evolution is actually not so far-fetched. Evolution as a result of chance shows itself in this story as a friend and foe: it led to the development of humans, but also to cancer and drug resistance. Using ASR to solve a modern cancer drugs mechanism is unorthodox, since until recently this method has been applied to recapitulate nature's paths to modern proteins with differential functions. Arguably the most famous ASR story has come from the Thornton lab in their successful inference of ancient corticoid receptors (Thornton et al., 2003; Ortlund et al., 2007; Bridgham et al., 2009). A story spanning over a half a dozen research papers not only shed light on the understanding of the different selectivity of modern steroid receptors for their corresponding hormones, but also answered some long standing questions in the field, including the role of epistasis in macromolecular evolution. Recently, ASR has leaped over to successfully recreate ancestral enzymes with reaction efficiencies near that of modern day enzymes (Perez-Jimenez et al., 2011; Hobbs et al., 2012; Ingles-Prieto et al., 2013; Risso et al., 2013; Boucher et al., 2014). Resurrection of enzymes has been an important step in validating the accuracy of ASR because of the need to maintain enzymatic activity, which is extremely sensitive to mutational change. These studies have largely focused on understanding changes in the enzyme's melting temperatures or underlying structural changes. The Gleevec story takes it to the next step to characterize the evolution of energy landscapes that ultimately underlies function.

## “Tell us what the future holds, so we may know that you are gods” (Isaiah 41, 23)

How can understanding of Gleevec selectivity and the differential *evolution* of kinases guide the *creation* of better cancer drugs? We are not god, we do not have the ultimate answer. However the door to intelligent design of successful cancer therapeutics may have opened a little wider with recent advances in genome information including ASR and personal genomic profiling, characterization of free energy landscapes of the drug binding process to targets, advances in medicinal chemistry and computation.

The history of Gleevec research teaches us a number of lessons: First, the correct microscopic binding model (meaning the correct scheme), ideally with quantification of each step, is crucial. Slow progress in understanding Gleevec's selectivity was in large extent due to the overwhelming attention to the DFG-loop conformational selection model (Cowan-Jacob et al., 2005; Dar et al., 2008; Shan et al., 2009; Aleksandrov and Simonson, 2010; Lovera et al., 2012; Lin and Roux, 2013; Lin et al., 2014). Second, the physical binding step that has been the major focus in docking simulations is only one piece of the puzzle, and conformational changes are crucially linked to both affinity and selectivity (Figure 2C). Therefore, experimental and computational efforts should be more centered on the dynamics of the target and drug/target complex. Third, the trivial (simple laws of thermodynamics) but at the same time profound recognition that conformational change after binding (an induced fit step) delivers two essential components of a good drug: increased affinity and long drug residence times on the target (Figure 2C). In addition, it can provide excellent specificity particularly when such conformational changes involve elements remote from the binding site as seen in Abl-Gleevec. In contrast, conformational selection (ability of the apo protein to sample multiple conformations) by definition weakens the overall drug affinity by the fraction of the protein in the binding-incompetent states. While such a step can offer drug specificity, the new results suggest that DFG-loop conformational selection seems to play only a minor role for kinase selectivity due to the fact that the DFG-loop readily interconverts between states. We propose that induced fit steps are in play in many successful drugs leading to very tight binding and long on-target residence times. Finally, molecular dynamics simulations will play an increasing role in rational drug design, but such simulations need to be based on the solid foundation of biochemical research. In the case of Gleevec and other kinase inhibitors, future computational emphasize should be centered on dynamics of the enzyme/drug complex characterizing the induced fit step and not on the DFG-loop dynamics. Having the correct binding scheme established with corresponding structural information available, MD can sample the conformational space identifying new local minima and potentially cryptic or allosteric sites that are hard to trap experimentally if they are low-populated. If such states are unique for a particular kinase, they can be excellent targets for new specific inhibitors.

We are excited about the future prospect of a happy marriage between experiments and computation, and between basic academic research and pharmaceutical industry to tackle the very challenging but rewarding goal of designing perfect weapons against deadly diseases.

### Conflict of interest statement

DK is the inventor on a patent applied for by Brandeis University that describes a biophysical platform for drug development based on energy landscapes. The authors declare that the research was conducted in the absence of any commercial or financial relationships that could be construed as a potential conflict of interest.
